# Investor sentiment and stock portfolio construction: A study based on the black-litterman model

**DOI:** 10.1371/journal.pone.0358348

**Published:** 2026-09-15

**Authors:** Linan Sun, Boyang Fu, Wenwen Liu

**Affiliations:** 1 College of Urban and Rural Planning, Henan University of Economics and Law, Zhengzhou, PR China; 2 School of Economics, Xihua University, Chengdu, Sichuan, PR China; Central University of Finance and Economics, CHINA

## Abstract

This study integrates the Black-Litterman (BL) model with deep learning techniques to construct a stock portfolio that incorporates investor sentiment. We develop a weighted investor sentiment index using comment data from the Eastmoney Stock Bar for eight representative stocks in the SSE 50 Index. The sentiment index is constructed through both a dictionary-based approach and the BERT model. Long Short-Term Memory (LSTM) networks are then employed to predict stock returns, which are incorporated as investor views into the BL framework for portfolio optimization. Empirical results demonstrate that incorporating investor sentiment significantly enhances stock price prediction accuracy, and the BERT-based sentiment index achieves the lowest prediction error. Within the BL model, the portfolio integrating the BERT sentiment index (BERT_BL) achieves an annualized return of 106.58% during the backtesting period, along with superior risk-adjusted performance metrics: a Sharpe ratio of 2.64, and a Sortino ratio of 4.18. The model remains robust even after accounting for transaction costs, parameter adjustments, and across different market environments. This study validates the application of textual sentiment analysis in quantitative investment, offering a methodological innovation by integrating unstructured data with classical asset allocation models. The findings provide practical insights for investors seeking to optimize portfolio management strategies.

## Introduction

In the field of financial investment, asset allocation is one of the core issues of investment management. As the cornerstone of portfolio optimization, the traditional mean-variance (MV) model suffers from inherent drawbacks including excessive asset position concentration, poor expected return predictability, and extreme sensitivity to input parameters, which greatly limit its practical applicability in real investment scenarios [1]. To address these deficiencies, Black and Litterman [2] proposed the classic BL model, which integrates Bayesian methods with investors’ subjective views. On this basis, the introduction of deep learning techniques further optimizes and automates the subjective judgment construction process in the BL model, effectively overcoming the manual operation defects of traditional models and improving the scientificity and practicality of portfolio allocation.

Among all asset classes, stocks are favored by a wide range of investors due to their high liquidity, high risk, and high return potential. As of 2023, the total market capitalization of China's A-share market has exceeded $10 trillion, making it the second-largest stock market globally, following the United States. This growth not only reflects the increasing economic strength of China but also demonstrates both domestic and international investors’ confidence in and participation in the Chinese market. With the advancement of internet and communication technologies, an increasing number of online platforms have become mediums for investors to express opinions and exchange information. Stock investors, who typically have limited access to information, are prone to engage in learning, mimicking behaviors, and emotional decision-making when participating in online social platforms that are influenced by public opinion. Consequently, financial textual information has been found to affect public investment choices and is one of the significant causes of stock market volatility [3–6].

Despite the vast amount of unstructured textual data containing investor sentiment generated in the social media era, existing BL frameworks have failed to effectively incorporate this information. Currently, most studies construct investor sentiment merely to enhance prediction accuracy, lacking integration with portfolio models. Such research overemphasizes theoretical value while ignoring practical applicability, and comparative investigations across different sentiment analysis models remain scarce. This limitation not only reduces the model's responsiveness to market sentiment but also neglects the impact of psychological factors on asset pricing as revealed by behavioral finance. Leveraging machine learning and artificial intelligence technologies, this study explores how to integrate sentiment analysis with the BL model to construct an effective stock portfolio, thereby enriching research in this field and helping investors participate in the long-term stable growth dividends of high-quality companies.

Firstly, we select eight representative constituent stocks from different industries within the SSE A50 index. We then employ web scraping technology to collect stock comments from the Eastmoney Stock Bar, spanning the period from July 1, 2022, to December 31, 2024. Based on these comments, we construct an investor sentiment index. In this paper, both a dictionary-based method and the BERT model [7] are used to construct daily investor sentiment indices, with weighting applied according to the number of views and secondary comments for each stock review.

Upon obtaining investor sentiment indices through both methods, we utilize LSTM networks [8] to predict future stock prices. In addition, a control group is created without considering investor sentiment in the price prediction. The results show that incorporating investor sentiment improves stock price prediction accuracy, with the BERT-based sentiment model yielding the smallest prediction error. Finally, we backtest the stock return predictions for all three scenarios within the BL model framework,comparing the results with benchmark models such as the equal-weighted model and the mean-variance model. The results demonstrate that the proposed BERT_BL model achieves the highest investment returns and optimal financial metrics. Even after accounting for transaction costs, adjusting the weights of subjective views in the BL model, and evaluating the model’s performance across different market regimes, the results remain robust.

The remainder of the paper is organized as follows. Related Work reviews the relevant literature, and Model Introduction presents the MV and BL models. Financial Text Mining and Sentiment Analysis describes the data and sentiment analysis methods, while Stock Return Prediction presents the descriptive statistics and return prediction approach. Empirical Analysis reports and discusses the empirical results. Research Expansion examines transaction costs and robustness, and Conclusion summarizes the main findings.

## Related work

This literature review is primarily divided into two sections: the first discusses the impact of investor sentiment on the stock market and its role in stock return prediction, and the second focuses on the integration of investor sentiment within the BL model framework for portfolio construction by scholars from both domestic and international perspectives.

### Research on the influence of investor sentiment

Existing research has demonstrated that investor sentiment has a significant impact on the stock market. For example, investor sentiment affects stock prices [9–12], volatility [13–16], and liquidity [17,18], leading to systematic and significant effects on stock market returns [13,19,20].

Investor sentiment can enhance the prediction accuracy of stock returns [21].incorporated company-specific topics and related sentiment into stock prediction models and conducted large-scale experiments to evaluate the role of sentiment analysis in stock forecasting tasks. The results indicated that the prediction accuracy for 18 stocks improved by 2.07% compared to using only historical prices, with the accuracy for more difficult-to-predict stocks increasing by 9.83% [22]. applied various machine learning and deep learning techniques, including support vector machines and naive Bayes, to improve the stability of stock price predictions by incorporating investor sentiment and other factors such as news [23]. scraped posts from the Eastmoney forum and combined the sparrow search algorithm with LSTM to predict future stock prices. Their results showed that adding sentiment indices could improve the model’s predictive ability, but they also suggested that the Chinese stock market is more suitable for short-term predictions. In addition, other scholars have verified the indispensable role of investor sentiment in stock price prediction using various methods [24–27].

### Research on portfolio construction using the BL model

The BL model has attracted considerable scholarly attention both domestically and internationally since its inception, particularly in the context of model research and refinement. Scholars have posited that the optimal portfolio in the BL model represents a combination of the market equilibrium portfolio and a weighted portfolio reflecting investors’ views. The weights are adjusted based on the optimism level of these views and the degree of investor confidence. Additionally, researchers have proposed methodologies to quantify investor confidence levels, thereby further enhancing the model's practical applicability [28,29].

Accurately and reasonably capturing investors’ subjective expectations is a critical aspect of the BL model. Some scholars have employed historical data to manually set subjective expectations as posterior information. For instance [30], constructed investors’ expectations by integrating high-frequency data, including news sentiment, accounting variables, and financial analysts’ recommendations. Compared to the market portfolio, the results highlighted the model's significant potential. Similarly [31], utilized the consensus target price from the Wind financial terminal as a proxy for investors’ views on individual stock returns. By incorporating this into an enhanced BL model, they investigated the improved strategy for the Shanghai Stock Exchange 50 Index under new asset regulations. Their findings demonstrated that the strategy could generate excess returns under relatively stable market conditions while also improving key risk-return metrics, such as the Sharpe ratio and information ratio.

Due to the inherent irrationality and uncertainty associated with manually set subjective expectations, some scholars have shifted to traditional econometric models to construct investors’ views. For example, numerous scholars have conducted predictions based on the GARCH model [32–34]. In another study, [35] utilized publicly available stock data from Yahoo Finance and applied the Autoregressive Integrated Moving Average (ARIMA) model to forecast stock prices. Their results indicated that the ARIMA model achieved high accuracy in short-term stock price predictions.

With the rapid advancement of computer technology and the exponential growth of data, researchers have increasingly adopted machine learning and artificial intelligence techniques to enhance portfolio performance [36–42]. For instance [43], proposed a dynamic asset allocation strategy that integrates LSTM-based return predictions with the BL model to optimize portfolio weights across MSCI industry indices. Empirical results demonstrate that this strategy outperforms traditional benchmarks in most holding periods, delivering superior returns and enhanced robustness [44]. proposed a method that combines LSTM networks with Support Vector Regression (SVR) to generate investor views, which are then incorporated into the BL model for portfolio optimization. Empirical results demonstrate that this approach outperforms the traditional buy-and-hold strategy in both emerging and developed markets. Furthermore, in research focused on constructing investor sentiment indices using the BERT model [45], used the FineBERT model to extract sentiment from Financial Times articles and integrated it with Monte Carlo simulations to improve stock return forecasts, using the results as views in the BL model. Similarly [46], applied BERT to news sentiment analysis and combined it with GRU, LSTM, and RNN for return prediction, finding that the BL-GRU model achieved the best risk-adjusted performance.

A review of the existing literature highlights the significant influence of investor sentiment on investment decisions and its critical role in enhancing the BL model's ability to construct effective portfolios. However, research integrating the BERT model within the BL framework for asset allocation remains limited, and no comparative analysis has been conducted with other investor sentiment models. The primary contribution of this study lies in constructing an investor sentiment index using both the dictionary method and the BERT model, based on stock forum comments, and employing Long Short-Term Memory (LSTM) networks to forecast stock returns. The findings demonstrate that incorporating investor sentiment significantly improves stock price forecasting accuracy, thereby enriching asset allocation methodologies within the BL model framework. Additionally, this research provides valuable insights into the application of machine learning and other artificial intelligence technologies in financial investment. The backtesting results, conducted using eight stocks from diverse industries within the SSE 50 Index, offer robust practical guidance for investors and practitioners.

## Model introduction

### BL model

The BL model addresses the challenge of directly estimating expected returns by combining market equilibrium returns with investor subjective views. Assuming market equilibrium, the prior expected excess returns are derived as $\Pi =\lambda \Sigma \omega_{eq}$, where $\omega_{eq}$ represents market capitalization weights. Investor views are incorporated as $P\mu =Q+\varepsilon_{p}$, where P is the pick matrix linking views to assets, Q is the view vector, and $\varepsilon_{P}\;N\left(0,\Omega \right)$. Applying Bayes’ theorem, the posterior expected returns and covariance matrix are:


$$\begin{array}{c} \mu^{*}={\left[(\tau \Sigma {)}^{-1}+P^{T}\Omega^{-1}P\right]}^{-1}\left[(\tau \Sigma {)}^{-1}\Pi +P^{T}\Omega^{-1}Q\right]\; \end{array}$$ (1)

$$\begin{array}{c} \Sigma^{*}=\Sigma +{\left[(\tau \Sigma {)}^{-1}+P^{T}\Omega^{-1}P\right]}^{-1} \end{array}$$ (2)

The optimal portfolio weights are then obtained as $\omega^{*}=\left(\Sigma^{*-1}\mu^{*}\right)/\lambda$.

## Financial text mining and sentiment analysis

### Data sources and processing

Eastmoney Stock Bar, an interactive community operated by Eastmoney Information Co., Ltd., serves as a prominent platform for stock investors to exchange investment insights. It is one of the most active communication hubs in the stock investment domain. This study utilizes Python-based web scraping technology to collect stock commentary from Eastmoney Stock Bar for eight stocks across different key industries within the SSE 50 Index, covering the period from July 1, 2022, to December 31, 2024. The selected stocks and their respective industries are as follows: Poly Developments (Real Estate), China Unicom, abbreviated as “CUCC” (Telecommunications), Hengrui Medicine (Pharmaceuticals), Yili Group (Food), China State Construction Engineering, abbreviated as “CSCEC” (Construction), Bank of China (Finance), Sinopec (Energy), and Advanced Micro-Fabrication Equipment Inc. China (Semiconductors), abbreviated as “AMEC.” These eight stocks are industry leaders, forming a diversified investment portfolio that includes cyclical industries (e.g., real estate and energy), defensive industries (e.g., pharmaceuticals and food), and growth-oriented sectors (e.g., telecommunications and semiconductors). This combination not only enhances return potential but also mitigates risk, making it well-suited for generating stable investment returns in complex market environments.

The preprocessing workflow is as follows: First, we scrape post titles, authors, page views, comment counts, and update times, and collect corresponding comment bodies to form complete valid comment samples matched with post titles. Given the arbitrariness, fragmentation, and severe noise interference of online textual data, we conduct elaborate and standardized preprocessing on the original corpus. Specifically, we eliminate 4,725 pieces of automatically released information from news agencies and official accounts, including news updates, annual report disclosures, and official announcements. Then, a total of 2,508 comments with missing values and complete duplicates are removed. Furthermore, considering that the original text contains numerous meaningless contents such as HTML tags, HTTP links, and escape characters, we adopt regular expressions to fully filter out these invalid characters. All cleaned texts are renumbered to form a standardized corpus applicable to subsequent sentiment analysis models, ultimately yielding 387,953 valid samples. Since this study adjusts the investment portfolio on a daily basis, the comments are segmented by day, with sentiment from the t−1 trading day treated as the sentiment period for the $t_{th}$ trading day. Additionally, sentiment generated on holidays or non-trading days is deferred to the next normal trading day.

As this study used publicly available online data and involved neither human participants nor animals, ethics committee approval was not required.

### Dictionary method

The dictionary method is one of the most traditional approaches in sentiment analysis. It does not rely on labeled training data; instead, it estimates the overall sentiment polarity of a text by counting sentiment-bearing words from a predefined sentiment dictionary. Compared with other sentiment analysis methods, it is simple to implement and computationally efficient. However, it is limited in handling long-range contextual dependencies and sarcasm. Due to its simplicity and efficiency, it is often regarded as a preferred approach for constructing straightforward sentiment indicators to predict stock price movements. For this study, two Chinese sentiment dictionaries are selected: the one proposed by Jiang et al. [47] and the Chinese Financial Sentiment Dictionary (CFSD). Both dictionaries are specifically tailored for the financial domain and are employed to quantify sentiment tendencies in text. To enhance the robustness and diversity of sentiment analysis, these two dictionaries are combined to create a more comprehensive sentiment lexicon. After merging and removing duplicate entries, the integrated lexicon contains a total of 9,661 words, including 3,551 positive words and 6,110 negative words.

The methodology for constructing the investor sentiment index using the dictionary method is as follows: First, the Jieba library is utilized to perform word segmentation on the input comments, converting them into word groups. Next, stop words are removed using the Harbin Institute of Technology (HIT) stop word list. Finally, sentiment scores are assigned based on the content of the comments. Specifically, the sentiment score increases by 1 for each occurrence of a positive word and decreases by 1 for each occurrence of a negative word. A higher sentiment score indicates a more positive sentiment.


$$\begin{array}{c} dic\left(k\right)=\frac{\sum_{i=1}^{N}Pos_{i}-\sum_{j=1}^{M}Neg_{j}}{N+M}\; \end{array}$$ (3)

Here, dic(k) represents the sentiment index of the $k_{th}$ comment using the dictionary method, $Pos_{i}$ denotes the $i_{th}$ positive word, and $Neg_{j}$ represents the $j_{th}$ negative word. N+M refers to the total number of positive and negative words.

### BERT

BERT [9] is a powerful pre-trained language model initially proposed by Google AI Research in October 2018. By utilizing a bidirectional Transformer architecture, it is capable of learning the contextual relationships of words within a sentence. Compared with the dictionary method and traditional machine learning sentiment analysis models, BERT significantly improves contextual understanding and is capable of constructing sentiment indices that exhibit stronger relationships with stock prices and returns. This study employs BERT to further examine the incremental contribution of advanced sentiment analysis models in the field of stock prediction. The model is pre-trained on large-scale unlabeled text data and then fine-tuned on a smaller amount of labeled data for specific tasks. This approach significantly reduces the need to collect large amounts of labeled data for each new task.

The pre-training stage of BERT consists of two important self-supervised tasks: Masked Language Modeling (MLM) and Next Sentence Prediction (NSP). MLM randomly masks 15% of the input tokens during the input stage, and the model predicts the masked tokens based on the surrounding contextual information, thereby learning bidirectional semantic representations within sentences. NSP is designed to determine whether two sentences are logically coherent and sequential, enabling the model to capture inter-sentence relationships.

This study adopts the BERT-base-Chinese pre-trained model as the core framework for the sentiment analysis task. The model contains 12 Transformer encoder layers, a hidden size of 768 dimensions, and 12 attention heads.

**Fig 1:**
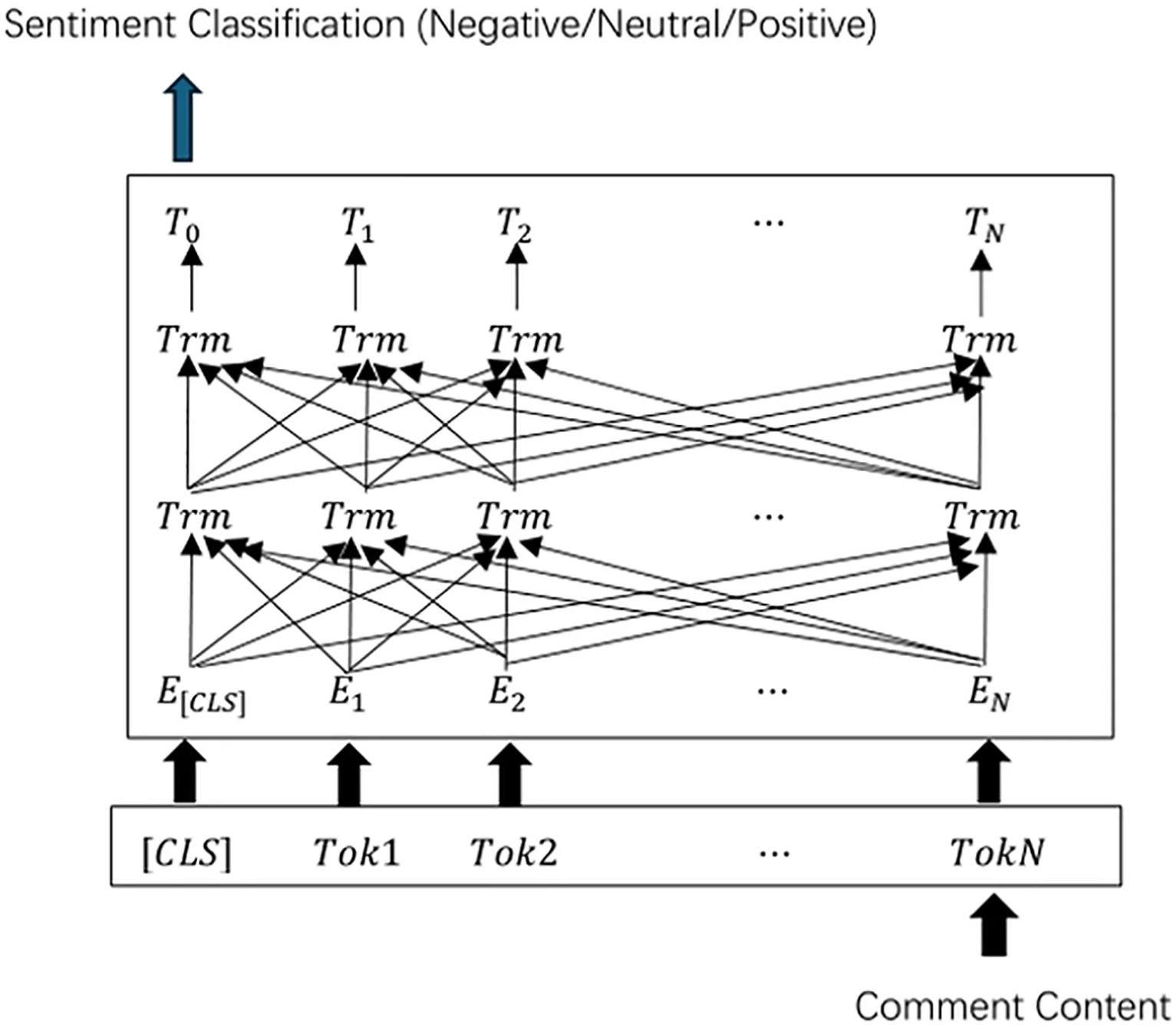
BERT model sentiment classification process.

Fig 1 illustrates the workflow of the BERT model for sentiment classification. First, the original review text is tokenized into a sequence of tokens through the Tokenizer and transformed into input representations by combining Token Embeddings, Positional Embeddings, and Segment Embeddings. Subsequently, the input sequence is fed into the BERT model composed of 12 Transformer encoder layers. Through structures such as Multi-Head Self-Attention, Feed Forward Networks (FNN), Residual Connections, and Layer Normalization, the model learns deep bidirectional semantic features of the text. Finally, the model extracts the 768-dimensional vector corresponding to the sequence-start token [CLS] and feeds it into a fully connected classification layer to categorize sentiments into three classes: negative, neutral, and positive. During training, the loss function and backpropagation algorithm are used to continuously optimize both the BERT parameters and the downstream classification layer.

To select an appropriate corpus for fine-tuning, this study utilizes a sentiment-labeled dataset of titles from Eastmoney Stock Bar related to the Huaxia SSE 50 ETF (available at https://huggingface.co), which has been manually annotated. The dataset is split into an 80% training set and a 20% test set. The dataset adopts a ternary labeling format, where 0 denotes negative sentiment, 1 represents neutral sentiment, and 2 indicates positive sentiment, with 3,170 negative entries, 3,024 neutral entries, and 4,452 positive entries respectively. Negative sentiment reflects a pessimistic outlook on a stock's rise or an optimistic view of its decline, while positive sentiment conveys the opposite. Neutral sentiment pertains to questions without subjective bias, content unrelated to stock movements, or comments expressing an indifferent view of stock fluctuations.

The optimal hyperparameters are determined through cross-validation and grid search. The detailed fine-tuning configurations of the BERT model are specified as follows. To ensure experimental reproducibility, the random seed is fixed at 42. The maximum sequence length of input text is set to 128, and the batch size is set to 16. The dropout layer adopts a dropout rate of 0.1, and the AdamW optimizer is employed for model optimization. A layered learning rate strategy is applied, where the learning rate for the BERT backbone is set to 2e-5, the learning rate for the classification head is set to 1e-4, and the weight decay coefficient is 0.01. A linear warm-up and decay learning rate scheduler is adopted, with the warm-up ratio accounting for 10% of the total training steps. The model is trained for 5 epochs with the cross-entropy loss function. Finally, the optimal model checkpoint is loaded to conduct performance evaluation on the test set. The results indicate an average classification accuracy of 74.69% on the test set. Subsequently, the 387,953 comments from the eight constituent stocks are input into the model for sentiment classification.

To assess the model's generalization capability on the scraped stock bar comments, this study randomly selects 1,000 comments from the entire dataset for manual annotation. These comments are categorized into negative, neutral, and positive sentiments based on the same criteria, followed by expert review. The fine-tuned model parameters are then applied to classify the comments. Table 1 displays the confusion matrix of the classification results, illustrating the comparison between the predicted and actual classifications generated by the BERT model. Based on the calculations, the overall classification accuracy is 52.3%, Specifically, the primary source of classification error does not stem from misclassification between positive and negative sentiments, but rather from confusion between polarized sentiments and neutral sentiment. In practice, the identification of neutral sentiment is both necessary and inherently controversial [48]. The probability of misclassifying negative comments as positive is extremely low, indicating that the fine-tuned BERT model is capable of effectively identifying and capturing sentiment expressions in investor comments. This capability is of considerable importance for the subsequent asset allocation process within the BL framework.

**Table 1:** Sentiment classification results.

		Actual Classification		
Predicted Classification		Negative	Neutral	Positive
Negative	275	36	31
Neutral	63	151	152
Positive	100	95	97

### Construction of the investor sentiment index

After conducting basic scoring and classification of the scraped comments using the dictionary method and the BERT model, the construction of the daily investor sentiment index also requires accounting for the influence of the comments. This influence is primarily reflected in the comment's view count and the number of secondary comments. Comments with higher view counts and more secondary comments are considered to have greater influence. Consequently, both factors are incorporated as weighting coefficients in the construction of the daily investor sentiment index. The methodology for constructing the investor sentiment index for the$i_{th}$ stock on the $t_{th}$ trading day is illustrated in the formula below:


$$\begin{array}{c} SI_{t}\left(i\right)=\frac{\sum_{n=1}^{N}SI_{t}\left(i,n\right)*\left(L_{t}\left(i,n\right)+P_{t}\left(i,n\right)\right)}{\sum_{n=1}^{N}\left(L_{t}\left(i,n\right)+P_{t}\left(i,n\right)\right)}\; \end{array}$$ (4)

**Fig 2:**
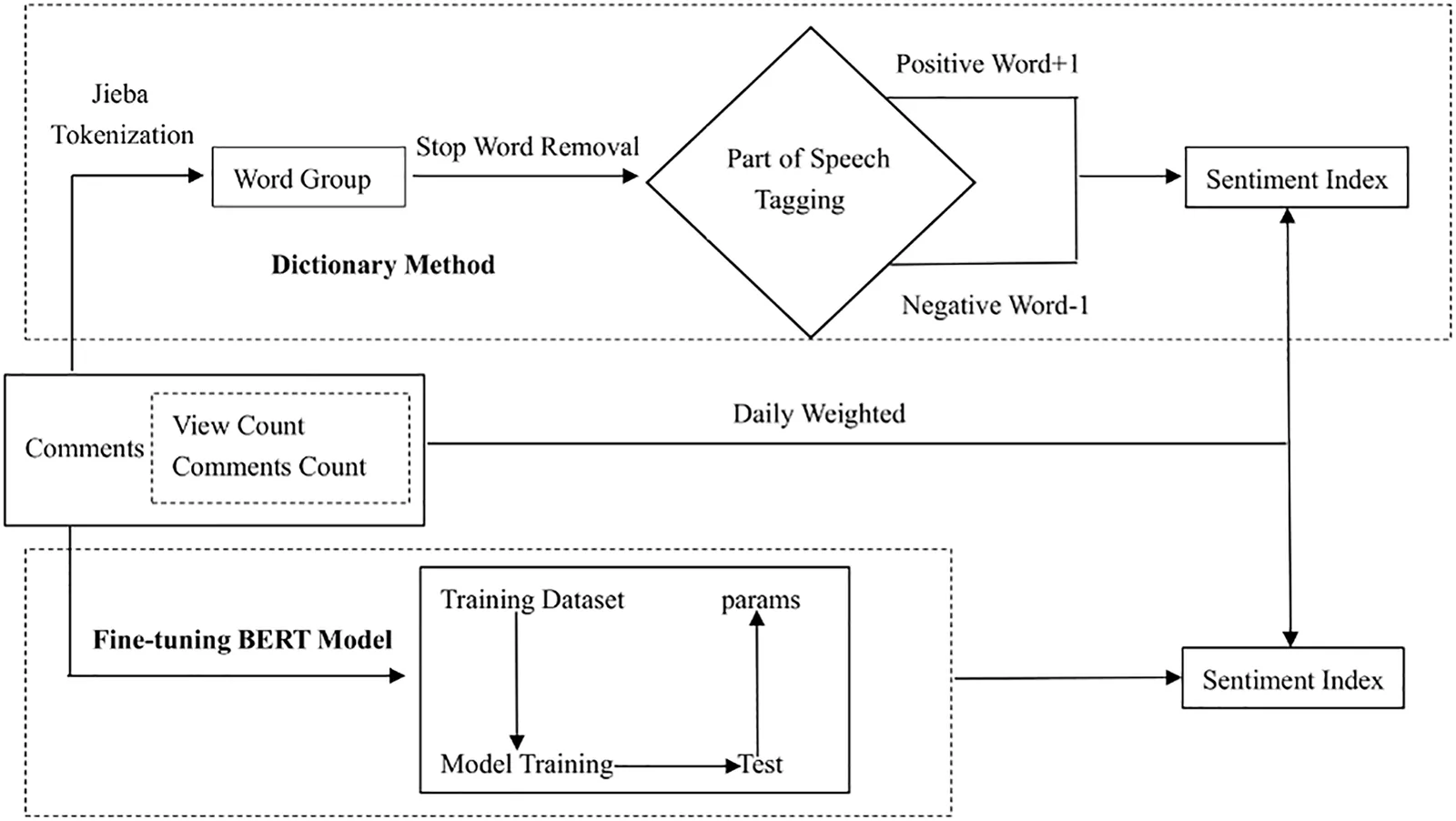
Investor sentiment index construction process.

Where $SI_{t}\left(i,n\right)$ represents the score or category of the $n_{th}$ comment for the $i_{th}$stock on the $t_{th}$ trading day, $L_{t}\left(i,n\right)$ denotes the view count of the corresponding comment, and $P_{t}\left(i,n\right)$ is the number of secondary comments associated with the comment. The final daily weighted investor sentiment index for the $i_{th}$ stock on the $t_{th}$ trading day is then obtained. The entire process for constructing the investor sentiment index is shown in Fig 2:

## Stock return prediction

This study selects eight stocks from diverse industries within the SSE 50 Index. Prior to predicting the future returns of these stocks, data on the opening price, highest price, lowest price, closing price, and trading volume for each stock from July 1, 2022, to December 31, 2024, are retrieved from the Wind database. By integrating sentiment indices derived from the dictionary-based approach and the BERT model, the dataset is divided into approximately 80% for the training set and 20% for the test set. The training set covers the period from July 1, 2022, to July 10, 2024, while the test set spans from July 11, 2024, to December 31, 2024. Stock prices for the test set are predicted, and the return rates are subsequently calculated. The return rate employed in this study is the logarithmic return:


$$\begin{array}{c} y_{t}=log\left(\frac{p_{t}}{p_{t-1}}\right) \end{array}$$ (5)

At this stage, we have completed the data collection and investor sentiment construction required for predicting stock returns. Table 2 presents the descriptive statistics of stock bar comments, price information, and investor sentiment indicators used for return prediction.

**Table 2:** Descriptive statistics of the data.

	Sinopec	Poly Developments	CUCC	Hengrui Medicine	Yili Group	CSCEC	Bank of China	AMEC
Average Comments	45	62	80	72	72	39	29	26
Highest Comments	468	463	1120	1330	2083	226	1157	208
Lowest Comments	1	1	1	1	1	1	1	1
Total Comments	40990	56719	73385	65406	65690	35873	26642	23248
Average Price (CNY)	5.32	11.67	4.49	42.85	27.38	5.22	3.74	142.75
Average Return Rate	0.10%	0.10%	0.08%	0.04%	0.03%	0.03%	0.12%	0.08%
Highest Price (CNY)	7.02	17.37	5.85	56.01	35.55	6.54	5.53	246.30
Highest Return Rate	7.63%	9.63%	9.65%	9.51%	8.12%	8.27%	9.38%	18.23%
Lowest Price (CNY)	3.47	7.45	3.07	31.63	21.37	4.32	2.69	79.85
Lowest Return Rate	−6.47%	−10.55%	−10.11%	−9.56%	−10.58%	9.69%	−5.79%	−21.57%
Average Sentiment (Dictionary)								
*unweighted*	0.25	0.20	0.20	0.20	0.16	0.15	0.26	0.34
*weighted*	0.92	0.87	0.51	1.44	0.43	0.50	0.47	1.02
Average Sentiment (BERT)								
*unweighted*	1.23	1.15	1.11	1.26	1.18	1.13	1.10	1.20
*weighted*	1.39	1.16	1.22	1.23	1.19	1.22	1.35	1.18
Highest Sentiment (Dictionary)								
*unweighted*	3.00	19.00	25.50	15.40	4.00	4.33	5.25	7.00
*weighted*	37.50	24.54	49.06	24.22	23.36	14.00	28.40	48.20
Highest Sentiment (BERT)								
*unweighted*	2.00	2.00	2.00	2.00	2.00	2.00	2.00	2.00
*weighted*	2.00	2.00	2.00	2.00	2.00	2.00	2.00	2.00
Lowest Sentiment (Dictionary)								
*unweighted*	−1.67	−1.80	−2.25	−2.67	−3.29	−6.00	−3.00	−5.00
*weighted*	−1.41	−2.25	−2.71	−3.30	−1.94	−4.96	−4.57	−8.10
Lowest Sentiment (BERT)								
*unweighted*	0.00	0.00	0.00	0.00	0.00	0.00	0.00	0.00
*weighted*	0.00	0.00	0.00	0.00	0.00	0.00	0.00	0.00
Sentiment-Return Correlation (Dictionary)	−0.0504	0.0496	0.1450^***^	0.0413	0.0444	0.2016^***^	0.0415	−0.0136
Sentiment-Return Correlation (BERT)	−0.0519	−0.0385	0.1668^***^	0.1107^***^	0.1231^***^	0.1633^***^	0.0553	0.1190^***^

Note: Sentiment–Return Correlation (Dictionary) denotes the Spearman correlation coefficient between the sentiment index constructed using the dictionary-based method and logarithmic stock returns. Sentiment–Return Correlation (BERT) denotes the Spearman correlation coefficient between the sentiment index constructed using the BERT-based method and logarithmic stock returns. *, **, and *** indicate statistical significance at the 10%, 5%, and 1% levels, respectively

The distribution of stock commentary data indicates substantial variation in investor attention across different stocks, with the total number of comments ranging from 23,248–73,385. A further examination reveals considerable fluctuations in the daily volume of comments for each stock. On some trading days, the number of comments falls to single digits, whereas on others it may surge to several hundred or even thousands. This pattern suggests that investor sentiment is often released intensively in response to unexpected events or information shocks.

After applying the weighting scheme, the average sentiment scores generated by the dictionary-based method increased from 0.15–0.34 in the unweighted setting to 0.43–1.44, while the maximum values rose from 3.00–25.50 to 14.00–49.06. In contrast, the minimum values changed only marginally. For the BERT-based method, the average sentiment scores increased slightly from 1.10–1.26 to 1.16–1.39, whereas the maximum and minimum values remained unchanged. The differences in weighting effects between the two approaches can be largely attributed to their underlying mechanisms. The dictionary-based method generates continuous sentiment scores, providing greater scope for adjustment through weighting, whereas the BERT-based method relies on discrete sentiment classification, resulting in a more limited impact of weighting.

The results of the Spearman correlation analysis indicate that investor sentiment indices are generally positively correlated with stock returns. Notably, compared with the traditional dictionary-based sentiment index, the sentiment index generated by the BERT model exhibits stronger and more statistically significant correlations with stock returns. This finding provides robust empirical support for the rationality and effectiveness of incorporating sentiment indicators into stock return forecasting models.

### Long Short-Term Memory (LSTM)

Long Short-Term Memory (LSTM) is a specialized variant of recurrent neural networks (RNNs) designed to address the challenges of vanishing and exploding gradients encountered when processing long sequences of data. It has gained widespread adoption in time series prediction tasks [49–52]. The LSTM architecture primarily comprises three key components: the forget gate, the input gate, and the output gate.

#### Forget gate.

The forget gate takes as input the current time step's input and the previous time step's hidden state. It uses a Sigmoid activation function to determine the proportion of information to discard, thereby controlling which useful information remains in the memory cell and avoiding the accumulation of irrelevant data.


$$\begin{array}{c} f_{t}=\sigma \left(W_{f}\cdot \left[h_{t-1},x_{t}\right]+b_{f}\right) \end{array}$$ (6)

Where $f_{t}$ represents the output of the forget gate, with a range of [0,1], σ denotes the Sigmoid activation function, $h_{t-1}$ is the hidden state from the previous time step, $x_{t}$ is the input at the current time step, and $W_{f}$ and $b_{f}$ are the weight matrix and bias term, respectively.

#### Input gate.

The input gate decides which new information will be input into the memory cell. First, the Sigmoid activation function determines the proportion to update, and then the Tanh activation function creates new candidate information.


$$\begin{array}{c} i_{t}=\sigma \left(W_{i}\cdot \left[h_{t-1},x_{t}\right]+b_{i}\right) \end{array}$$ (7)

$$\begin{array}{c} {\tilde{C}}_{t}=tanh\left(W_{c}\cdot \left[h_{t-1},x_{t}\right]+b_{c}\right) \end{array}$$ (8)

$$\begin{array}{c} C_{t}=f_{t}*C_{t-1}+i_{t}*{\tilde{C}}_{t} \end{array}$$ (9)

Where $i_{t}$ represents the output of the input gate, with a range of [0,1], ${\tilde{C}}_{t}$ is the candidate memory at the current time step, with a range of [−1,1], and $W_{i}$, $W_{c}$, and $b_{i}$,$b_{c}$ are the weight matrices and bias terms, respectively.

#### Output gate.

The output gate determines which parts of the memory cell at the current time step should be output. It then uses the Tanh activation function to extract the hidden state, which is passed on to the next time step.


$$\begin{array}{c} o_{t}=\sigma \left(W_{o}\cdot \left[h_{t-1},x_{t}\right]+b_{o}\right) \end{array}$$ (10)

$$\begin{array}{c} h_{t}=o_{t}*tanh\left(C_{t}\right) \end{array}$$ (11)

Where $o_{t}$ represents the output of the output gate, with a range of [0,1], $h_{t}$ is the hidden state at the current time step, $C_{t}$ is the memory cell at the current time step, and $W_{o}$ and $b_{o}$ are the weight matrix and bias term of the output gate, respectively.

### Stock price prediction

In this study, the LSTM model is employed to predict stock prices by incorporating scaled inputs of the stock's opening price, highest price, lowest price, closing price, and trading volume at each time point. The model is evaluated under three distinct scenarios, each with varying levels of investor sentiment integration. The first scenario incorporates investor sentiment derived from the BERT model, the second scenario includes sentiment based on the dictionary method, and the third scenario excludes any investor sentiment entirely.

During model implementation, grid search is adopted to tune key hyperparameters so as to avoid biases caused by manual parameter setting. The ranges of candidate parameter combinations are presented in Table 3. Considering the unique characteristics of historical data for individual stocks, we train and select optimal configurations separately for each stock while maintaining a unified model structure, to obtain more targeted settings. All input variables are standardized before being fed into the model, which mitigates the impact of dimensional differences on training and improves convergence efficiency. The Adam algorithm is utilized for model optimization.

**Table 3:** Hyperparameters of the LSTM model.

Hyperparameter	Definition	Values
batch_size	Number of samples per training batch	16, 32, 64
lstm_units	Number of neurons in the LSTM layer	16, 32, 64
dense_units	Number of neurons in the dense layer	16, 32, 64
epochs	Number of training iterations	50, 100
dropout	Neuron dropout rate between dense layers	0.1, 0.2
time_window_size	Length of the time window	1, 5, 10

**Fig 3:**
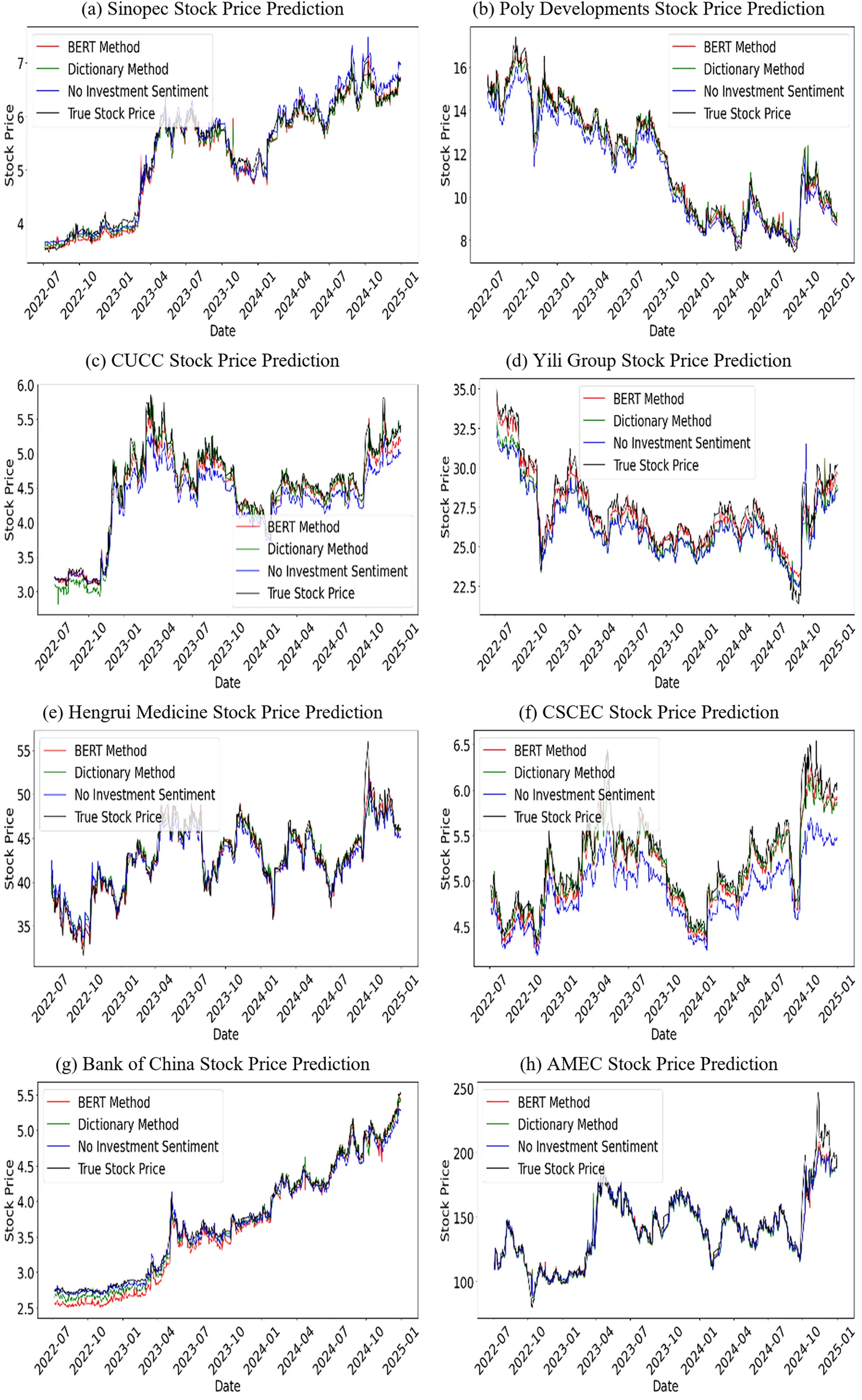
Stock price prediction results.

In the Fig 3, the black line represents the actual stock price, the red line denotes the predicted results based on investor sentiment derived from the BERT model, the green line indicates the predicted results based on sentiment from the dictionary method, and the blue line corresponds to the predictions without incorporating any investor sentiment. As illustrated in the Fig 3, the LSTM model effectively captures the trend of the stock price time series data. As training progresses, the model's fit to the training data improves. The three colored lines closely track the actual stock price line; however, the blue line (without investor sentiment) exhibits some deviation compared to the red and green lines (which incorporate investor sentiment). No overfitting is observed in the test set relative to the training set. Table 4 presents the Mean Squared Error (MSE) results for the test set under different investor sentiment scenarios.

**Table 4:** Stock price prediction mean square error.

MSE	Sinopec	Poly Developments	CUCC	Yili Group
**BERT**	0.0185	0.1893	0.0277	0.8229
**Dictionary**	0.0151	0.2059	0.0119	1.3485
**No Sentiment**	0.0766	0.2750	0.0929	1.4541
**MSE**	**Hengrui Medicine**	**CSCEC**	**Bank of China**	**AMEC**
**BERT**	1.2762	0.0316	0.0073	171.304
**Dictionary**	1.9880	0.0416	0.0076	188.731
**No Sentiment**	2.4172	0.2777	0.0173	200.990

Regardless of the investor sentiment method employed, the prediction error for the test set is consistently lower when investor sentiment is incorporated compared to the scenario without investor sentiment. This further validates that incorporating investor sentiment enhances stock price prediction performance. Additionally, with the exception of Sinopec and China Unicom, the investor sentiment predicted using the BERT model outperforms the sentiment predicted using the dictionary method for most stocks. This indicates that the investor sentiment index constructed by the BERT model provides a more accurate quantification of investor sentiment, enabling the LSTM model to generate more precise predictions of future stock movements.

## Empirical analysis

### BL model parameters

First, in this study, the LSTM model is employed to predict stock closing prices from July 11, 2024, to December 31, 2024, based on the opening price, highest price, lowest price, closing price, trading volume, and investor sentiment under different scenarios. These predictions are then used to calculate the logarithmic returns of the stocks. The investor views matrix and other parameters are integrated into the BL model to estimate the expected excess returns mean and covariance matrix of assets. By solving the optimization problem, the daily investment weights for each asset are derived. We assume that the investor starts with an initial asset value of 1 yuan, and assets are reallocated every trading day over the testing period, which spans 117 trading days.

In the BL model, investor views can be categorized into relative and absolute views. Absolute views refer to the investor's expectations of a specific stock’s excess return, for example, “Stock A is expected to generate a 3% excess return tomorrow.” Relative views, on the other hand, compare the expected returns of different stocks, such as, “Stock C is expected to generate 1% more excess return than Stock B tomorrow.” In this study, absolute views are adopted, and the excess returns predicted by the LSTM model are treated as subjective views. Therefore, the views covariance matrix p is an 8 × 8 identity matrix. As for the covariance matrix Σ, it is estimated through a rolling method starting from July 11, 2024, using the historical excess returns of the past 200 trading days until December 31, 2024. The error term in the covariance matrix is set as $\Omega =\tau *P\Sigma P^{T}$, where τreflects the proportion of the investor's subjective views, and λ is the investor's risk aversion coefficient. In this study, following Idzorek [29], we assume τ=0.025, $\lambda =\text{E}\left(r_{m}\right)-r_{f}/\sigma_{m}^{\text{2}}$, where the market risk-free rate $r_{f}$ is taken as 2.39% (the average yield to maturity of China's 10-year government bonds in 2024), converted into daily logarithmic returns. The number of trading days per year is assumed to be 252. The specific parameters of the BL model are shown in the Table 5:

**Table 5:** BL model parameters and values.

params	Description	Value
p	Views Covariance Matrix	8 × 8 Identity Matrix
Q	Investor Views Matrix	Expected Excess Returns calculated using LSTM predictions
Σ	Covariance Matrix	Rolling calculation based on excess returns from the past 200 trading days
Π	Implied Excess Return	$\Pi =\lambda \Sigma \omega_{eq}$
Ω	Covariance Matrix Error	$\Omega =\tau *P\Sigma P^{T}$,a diagonal Matrix
$r_{f}$	Risk-Free Rate	10-year Chinese Government Bond Average Yield to Maturity
τ	Proportion of Investor Subjective Views	τ=0.025

### Results evaluation

This study sequentially constructs investment portfolios for stocks using investor sentiment derived from the BERT model, dictionary method, and without any sentiment incorporation, within the BL model framework. To further compare the constructed portfolio returns, a series of benchmark models were selected for comparison.

1. Equal_Weights: The eight constituent stocks are equally weighted and held until the end of the evaluation period.
2. SSE_50: The Shanghai Stock Exchange 50 Index, which reflects the overall performance of the 50 most representative stocks in the Shanghai Securities Market, based on large market capitalization and high liquidity.
3. MV: The traditional mean-variance model, where stock expected returns and volatility are calculated based on the past 200 trading days.
4. Market_Value_Weights: The daily investment weights are calculated based on the market capitalization of each stock.

The benchmark models adopted in this study establish a comprehensive and representative framework for comparative analysis. As a simplistic and neutral reference benchmark, the Equal_Weights model operates independent of market data and theoretical assumptions. The SSE_50 index captures the performance of Chinese blue-chip stocks, serving as a universally acknowledged market benchmark. In contrast, the MV model embodies a classic, theoretically optimal investment strategy derived from historical risk-return trade-off dynamics. Lastly, the Market_Value_Weights model replicates real-world passive investment practices widely applied in index funds and ETFs. Collectively, these benchmarks cover a broad spectrum of investment philosophies, ranging from passive and practical approaches to active and theoretical strategies. This multi-dimensional framework allows for a thorough and robust evaluation of the proposed sentiment-integrated portfolio strategies.

To provide a more comprehensive evaluation of the performance of these strategies, in addition to using cumulative logarithmic returns as the primary metric, the study also incorporates the following financial indicators:

1) Annualized Return: This metric measures the compounded average return of the portfolio per year.

$$\begin{array}{c} AR={\left(\prod (1+r)\right)}^{\frac{252}{N}}-1 \end{array}$$ (12)

Where r is the daily logarithmic return, and N represents the length of the backtest period in days.

2) Sharpe Ratio: Proposed by Sharpe [53], this ratio describes how much excess return is gained per unit of risk taken.

$$\begin{array}{c} Sh.R=\frac{E\left(R_{p}\right)-r_{f}}{\sigma_{p}} \end{array}$$ (13)

Where $E\left(R_{p}\right)$ is the portfolio's annualized return, $\sigma_{p}$ is the standard deviation of the portfolio, and $r_{f}$ is the annualized risk-free rate.

3) Sortino Ratio: Proposed by Sortino and Price [54], this ratio measures how much excess return is gained per unit of downside risk.

$$\begin{array}{c} Sr.R=\frac{E\left(R_{p}\right)-r_{f}}{\sigma_{d}} \end{array}$$ (14)

Where $\sigma_{d}$ represents the downside standard deviation.

4) Maximum Drawdown: This metric calculates the largest possible loss during the evaluation period.

$$\begin{array}{c} MDD={max}_{0<t<d}\frac{Value_{t}-Value_{d}}{Value_{t}} \end{array}$$ (15)

Where $Value_{t}$ represents the highest net value during the backtest, $Value_{d}$ represents the lowest value after the maximum value has occurred.

**Fig 4:**
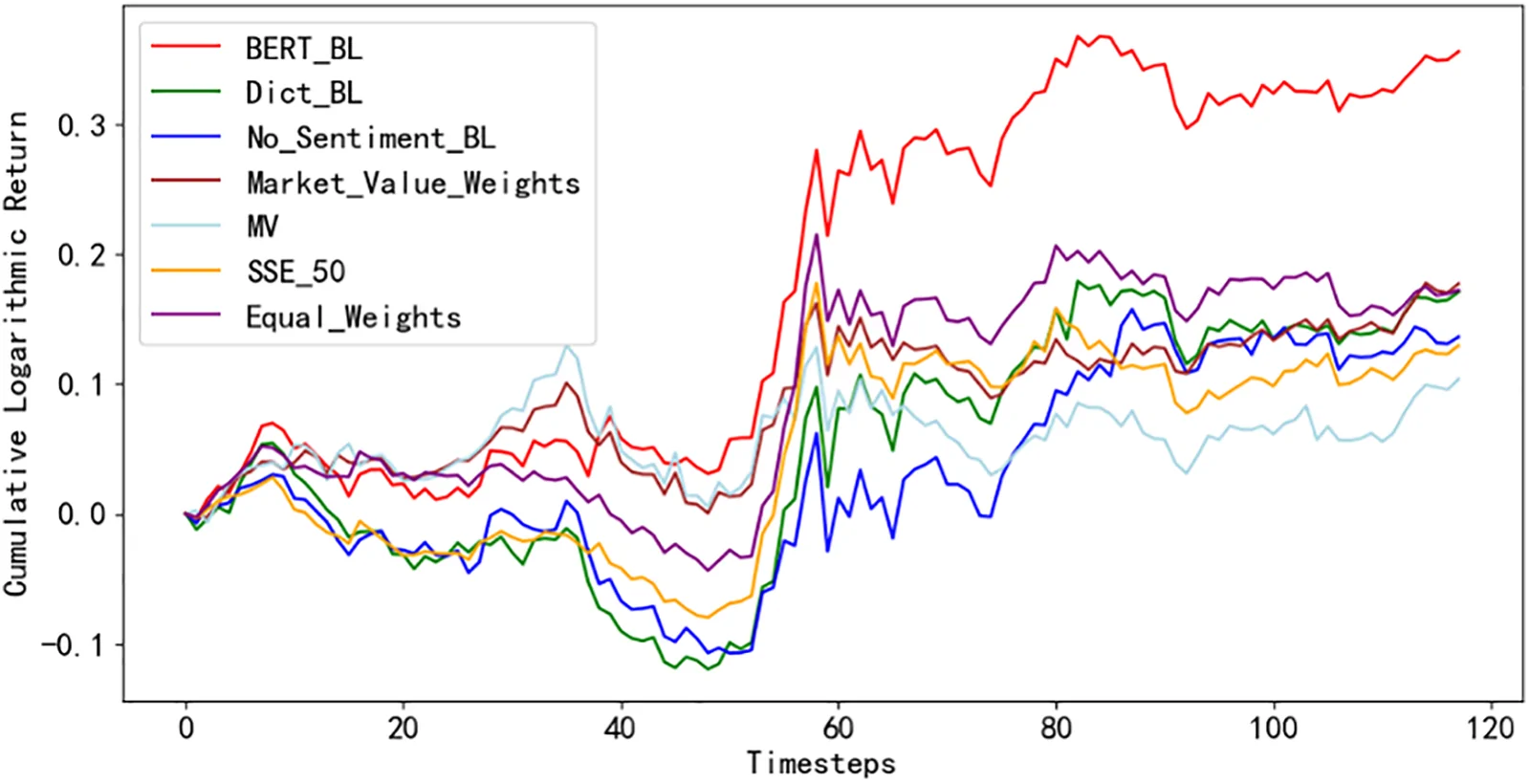
Cumulative logarithmic returns of investment strategies.

From Fig 4, it is evident that the investment returns of the various strategies were relatively similar at the beginning of the backtest period. However, as the investment process progressed, significant divergences emerged. In the early stages of the backtest, the Market_Value_Weights and MV models demonstrated the strongest performance. The MV model even temporarily outperformed all other strategies but was soon surpassed by BERT_BL. This further underscores that while the MV model can generate a robust investment portfolio, its performance is unstable due to excessively concentrated weights, as reflected in its highest maximum drawdown. Among the three BL models, the portfolio constructed using BERT_BL outperformed both Dict_BL and No_Sentiment_BL. Dict_BL and No_Sentiment_BL portfolios underperformed most benchmark models during the majority of the backtest period, highlighting the importance of incorporating accurate investor views in the BL framework.

In the later stages of the backtest, all models exhibited a significant upward trend in returns. This can be attributed to a series of positive market stimuli introduced by China starting in late September 2024. Market sentiment improved, and expectations for policy support fueled a market rally, followed by a broad-based market correction. Among all the BL models, the BERT_BL model delivered the best performance. This is primarily because BERT is more sensitive to sudden shifts in market sentiment, capturing these changes more effectively than the Dict_BL model. Although the Dict_BL model also achieved significant returns, the sentiment derived from the dictionary-based method lagged behind market shifts, resulting in less accurate stock price predictions and an inability to construct a more efficient portfolio. The No_Sentiment_BL model performed the worst, showing only a marginal rise in returns during the final stages and underperforming most benchmark models. This further validates that incorporating investor sentiment enhances model performance, with the BERT_BL model excelling in capturing changes in market sentiment.

From the perspective of financial indicators shown in Table 6, BERT_BL and Dict_BL exhibited higher annualized returns, Sharpe ratios, and Sortino ratios compared to No_Sentiment_BL, indicating that incorporating investor sentiment leads to higher returns without increasing risk.

**Table 6:** Summary of financial indicators for investment models (excluding transaction costs).

Model	AR(%)	Sh.R	Sr.R	MDD(%)
BERT_BL	106.58%	2.64	4.18	6.84%
Dict_BL	38.66%	1.21	1.85	6.16%
No_Sentiment_BL	28.60%	0.94	1.24	4.78%
Market_Value_Weights	43.28%	1.74	2.42	0.75%
MV	21.40%	0.83	1.10	11.66%
SSE_50	28.51%	1.09	1.67	9.50%
Equal_Weights	40.41%	1.40	2.16	8.21%

Maximum drawdown (MDD) is a core risk indicator for real-world portfolio management, as it quantifies the maximum peak-to-trough loss during the investment period and directly reflects the downside risk and anti-crash capability of investment strategies under extreme market fluctuations. The traditional MV model exhibits the highest MDD of 11.66%, indicating severe downside risk and poor stability in practical investment, which further verifies the inherent defect of the mean-variance framework in over-pursuing theoretical returns while ignoring extreme risk exposure. Because the BL model integrates investor subjective views with market equilibrium returns. When sentiment signals are strong, it may significantly adjust portfolio weights to pursue higher returns. This aggressive allocation strategy tends to perform well during market uptrends but can lead to larger drawdowns if the market reverses [55]. The Market_Value_Weights model has the lowest maximum drawdown, likely because the higher weight allocated to large-cap stocks contributes to greater stability, which helps reduce the overall volatility of the portfolio.

In summary, incorporating investor sentiment enhances stock price prediction accuracy, and the BERT model provides a more effective quantification of market sentiment. Consequently, the BL model with BERT-derived sentiment and stock returns as investor viewpoints outperforms other models, delivering higher and more stable returns.

## Research expansion

### Transaction costs

All investment strategies discussed in this study start with the standard academic assumption of zero transaction costs. Nevertheless, transaction costs are an indispensable factor in real-world active portfolio construction, as frequent rebalancing and position adjustment will continuously reduce net investment returns. For A-share stock trading, major transaction costs mainly consist of brokerage commissions and stamp duties. To make our backtesting results more consistent with practical investment operations, we set transaction fees in accordance with the prevailing trading rules of China’s stock market: a commission fee of 0.03% is charged for both stock purchases and sales, while a stamp duty of 0.1% is levied exclusively on sell orders. Specifically, if the weight of a stock on the current trading day exceeds that of the previous day, a commission of 0.03% is charged. Conversely, if the stock's weight is lower than the previous day, both the 0.03% commission and the 0.1% stamp duty are applied. The detailed formulas are as follows:


$$\begin{array}{c} cost=\left\{ \begin{array}{cc} @ll@0.03\%*\sum \left(\omega_{t}-\omega_{t-1}\right) & \omega_{t}>\omega_{t-1}\\ (0.03\%+0.1\%)*\sum \left(\omega_{t-1}-\omega_{t}\right) & \omega_{t-1}>\omega_{t} \end{array}\right. \end{array}$$ (16)

Where$\;\omega_{t}$represents the weight of each component stock on the current trading day, and $\omega_{t-1}$ represents the weight of each component stock on the previous trading day. For both the Equal Weights and SSE 50 Index strategies, a buy-and-hold approach is adopted, and transaction costs are not considered. Table 7 presents the financial metrics of each investment strategy after accounting for transaction costs:

**Table 7:** Summary of financial indicators for investment models (including transaction costs).

Model	AR(%)	Sh.R	Sr.R	MDD(%)
BERT_BL	51.87	1.55	2.47	8.23
Dict_BL	4.69	0.22	0.34	7.44
No_Sentiment_BL	−4.48	−0.10	−0.13	6.70
Market_Value_Weights	43.02	1.73	2.41	0.75
MV	18.75	0.74	0.98	11.76
SSE_50	28.51	1.09	1.67	9.50
Equal_Weights	40.41	1.40	2.16	8.21

The results show that transaction costs lead to a noticeable decline in returns across all BL model portfolios. Even under this realistic constraint, the BERT_BL model still achieves the best overall performance. The BL series models are designed for active asset allocation, which requires frequent rebalancing in practical portfolio operation; repeated position adjustments cause continuous accumulation of transaction costs and erode total returns. Nevertheless, the BERT_BL model retains the highest annualized return and Sortino ratio, which is consistent with the findings in Table 6. It demonstrates that the proposed model maintains robust competitiveness even when real transaction expenses are considered.

Different from active allocation strategies, the market-value-weighted portfolio shows little return loss after deducting transaction costs. In practical investment, market capitalization evolves slowly, so the daily adjustment range of stock weights is very limited. The low frequency and small scale of position changes keep transaction costs at a low level, which exerts almost no adverse impact on long-term portfolio returns.

### Robustness test

In the BL model, the parameter τ represents the weight assigned to the investor’s subjective views, which can significantly influence the model’s investment performance. When τ is small, investors place greater trust in market information, and the BL model tends to rely more heavily on prior information. Conversely, when τ is large, the model assigns more weight to the investor's subjective views. This section further examines whether the BERT_BL model can maintain optimal investment performance when τ is adjusted.

As shown in Table 8, this study investigates the sensitivity of the Black-Litterman model to changes in the parameter τ. The results indicate that whether the model places more weight on subjective views or market equilibrium returns, the portfolios constructed by the BERT_BL model maintain high returns and low volatility. The default setting of τ=0.025 yields a nearly optimal trade-off between return and risk, aligning with the commonly recommended value in existing literature. Moreover, the financial indicators of the BERT_BL model remain largely stable across different τ values and consistently outperform other Black-Litterman variants and benchmark models. These findings highlight the strong robustness and practical effectiveness of the BERT_BL model in portfolio construction and provide a reliable reference for parameter selection in real-world investment practices.

**Table 8:** Robustness test of parameter τ.

τ	AR(%)	Sh.R	Sr.R	MDD(%)
0.001	52.00%	1.55	2.48	8.22%
0.025	51.87%	1.55	2.47	8.23%
0.05	51.76%	1.55	2.47	8.23%
0.1	51.57%	1.54	2.46	8.24%
0.2	51.38%	1.53	2.45	8.26%
0.4	51.27%	1.52	2.45	8.28%
0.8	51.86%	1.52	2.45	8.31%
1	51.99%	1.52	2.45	8.32%

To further validate the robustness of the model, this study adopts the methodology proposed by [56] by extracting bull and bear market phases from the entire backtesting period, thereby examining the performance of various models under different market conditions. The identification of bull and bear market cycles is based on prominent and alternating local price extremes, which must meet specific magnitude and duration criteria to avoid mistaking minor fluctuations for genuine trend reversals.

Given that the eight selected stocks in this study are all constituents of the SSE 50 Index, the market environment is determined based on the price movements of the SSE 50 during the backtesting period. Specifically, at each time point, the algorithm checks whether the point represents the unique maximum or minimum within a surrounding time window. A local maximum is identified as a peak, and a local minimum as a trough. To ensure meaningful trend identification, the amplitude and duration between peaks and troughs must satisfy certain thresholds. Considering the relatively short backtesting interval in this study, and in order to reliably capture both bull and bear market periods without omission, peaks and troughs are defined as the highest and lowest values within a 15-day window, respectively. Moreover, the price change between a peak and a trough must be no less than 10%, and the transition must span at least 10 trading days.

As presented in Table 9, we split the backtesting sample into two typical market environments for robustness analysis: a bull market lasting 11 trading days and a bear market spanning 35 trading days. This division aligns with the stylized fact of China’s stock market, where bull markets are generally shorter in duration compared with prolonged bear markets. We then assess the performance of all competing models separately within these two market scenarios.

**Table 9:** Market regime classification results.

Market Regime	Period	Duration
Bull Market	2024.9.13-2024.10.08	11
Bear Market	2024.10.08-2024.11.25	35

**Fig 5:**
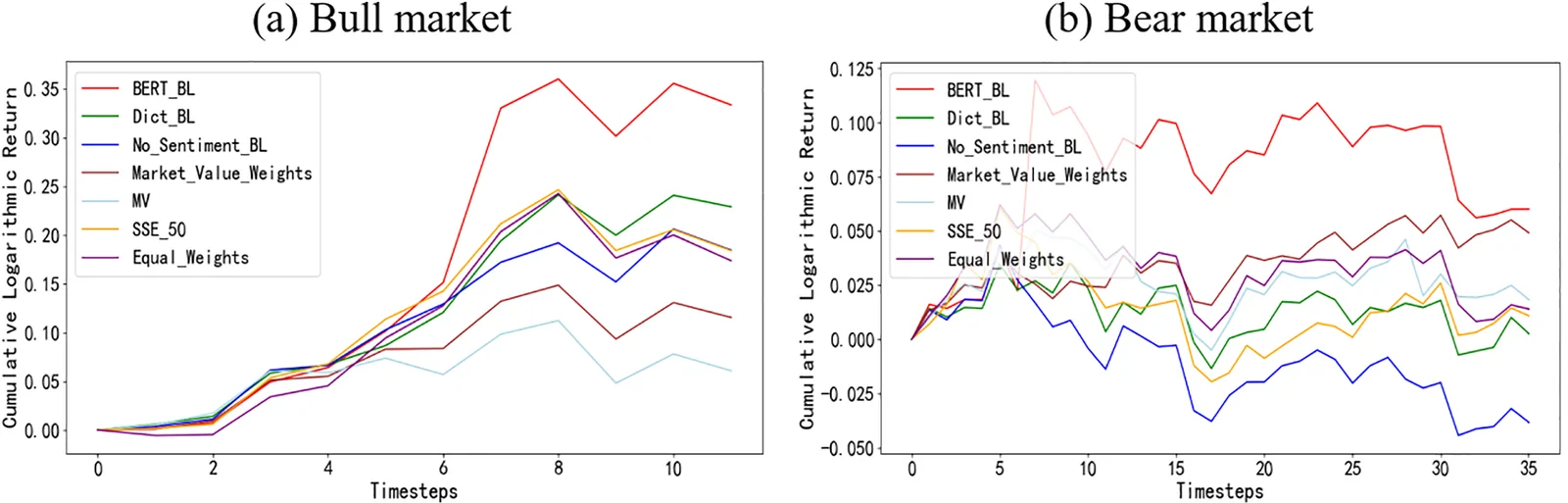
Cumulative logarithmic returns of investment strategies during bull/bear market.

Fig 5 compares the investment outcomes of different strategies across diverse market conditions. The results reveal that the BERT_BL strategy yields higher cumulative log returns than other BL model variants and the benchmark throughout the bull and bear periods. In bull markets, the portfolio based on BERT_BL effectively tracks upward price movements and maintains steady profit growth. During the bear market, despite a 10% drop in the SSE 50 Index, the proposed model still retains favorable return levels. Such outcomes demonstrate its reliable performance amid severe market fluctuations. Overall, the above results solidly validate the robustness of our model when applied to different market conditions.

## Conclusion

This study collected comments from Eastmoney Stock Bar for eight stocks across different industries within the SSE 50 Index, covering the period from July 1, 2022, to December 31, 2024. Investor sentiment indices were constructed using both the dictionary method and the BERT model, with daily weighting applied based on reading counts and secondary comments. Subsequently, LSTM was employed to predict future stock returns by integrating investor sentiment and price information, with the sentiment index serving as the investor's subjective view in the BL model. The results demonstrated that incorporating investor sentiment significantly enhanced stock price prediction accuracy, and among all BL models, only the BERT_BL model outperformed other benchmark models in terms of both returns and financial metrics during the backtest period, proving that BERT can accurately quantify investor sentiment and improve the rationality and accuracy of investors’ subjective expectations. Furthermore, the model's performance remained robust after accounting for transaction costs, altering model parameters, and across different market environments, thereby validating the model's effectiveness.

Based on the findings of this study, the following recommendations are proposed: Given the significant impact of investor sentiment on the market, regulatory bodies should implement more investor education initiatives to help investors recognize and respond rationally to market sentiment fluctuations. Simultaneously, improving the transparency of information disclosure by listed companies can reduce information asymmetry and foster a more informed investment environment. Furthermore, considering that sentiment-driven market fluctuations may exacerbate systemic risks, regulators should intensify the monitoring of social media platforms and investment forums to prevent the spread of false information and mitigate market manipulation.

Future research will address the key limitations of this study from multiple improvement perspectives. First, more advanced machine learning models will be employed for financial sentiment analysis and stock prediction to better capture complex market sentiment dynamics. Second, future work will critically examine the inherent biases of social media-based sentiment proxies. Eastmoney Stock Bar comments mainly reflect retail investor opinions and lack institutional views, while colloquial language, spam, and manipulated textual information may distort genuine sentiment signals. Meanwhile, further optimization is required to mitigate the domain adaptation and interpretability limitations of the generic BERT model in financial text mining. Third, to narrow the gap between backtesting and real-world investment, future simulations will incorporate practical trading constraints including slippage costs and portfolio weight limits, alongside liquidity restrictions and take-profit/stop-loss rules. We will also expand the investment universe to cover diverse domestic and international stocks.

## Supporting information

S1 Data Dataset used for empirical analysis. (ZIP)
